# Host-derived RANKL is responsible for osteolysis in a C4-2 human prostate cancer xenograft model of experimental bone metastases

**DOI:** 10.1186/1471-2407-7-148

**Published:** 2007-08-03

**Authors:** Colm Morrissey, Paul L Kostenuik, Lisha G Brown, Robert L Vessella, Eva Corey

**Affiliations:** 1 Department of Urology, University of Washington, Seattle, WA, USA; 2 Puget Sound VA Medical Center, Seattle, WA, USA; 3 Amgen Inc, Thousand Oaks, CA, USA

## Abstract

**Background:**

C4-2 prostate cancer (CaP) cells grown in mouse tibiae cause a mixed osteoblastic/osteolytic response with increases in osteoclast numbers and bone resorption. Administration of osteoprotegerin (OPG) blocks these increases, indicating the critical role of RANKL in osteolysis in this model. The objective of our study was to investigate whether RANKL expressed by tumor cells (human origin) directly stimulates osteolysis associated with the growth of these cells in bone or whether the increased osteolysis is caused by RANKL expressed by the host environment cells (murine origin). The relative contribution of tumor-*vs. *host-derived RANKL has been difficult to establish, even with human xenografts, because murine and human RANKL are both capable of stimulating osteolysis in mice, and the RANKL inhibitors used to date (OPG and RANK-Fc) inhibit human and murine RANKL.

**Methods:**

To address this question we used a neutralizing, antibody (huRANKL MAb), which specifically neutralizes the biological activities of human RANKL and thereby the contribution of C4-2 derived RANKL in this tibial injection model of experimental bone metastases.

**Results:**

Administration of huRANKL MAb did not inhibit the osteolytic response of the bone to these cells, or affect the establishment and growth of the C4-2 tumors in this environment.

**Conclusion:**

In conclusion, our results suggest that in this model, murine RANKL and not the tumor-derived human RANKL is the mediator of the osteolytic reaction associated with C4-2 growth in bone. We hypothesize that C4-2 cells express other factor/s inducing host production of RANKL, thereby driving tumor-associated osteolysis.

## Background

Prostate cancer (CaP) predominantly metastasizes to the bone, and bone metastases are the main cause of morbidity. While CaP metastases are usually osteoblastic in nature [1-3], there is an osteolytic component to the disease [4,5] which is manifested by increases in osteolytic markers in the serum and urine of patients with advanced CaP [6-8]. Increased bone resorption is also a prognostic factor for skeletal-related events in metastatic CaP [9]. However, how CaP cells induce an increased osteolytic reaction is not fully understood.

The RANKL/RANK/OPG system is critical in regulating osteoclastogenesis, and therefore is involved in bone remodeling (reviewed in [10]). Osteoclastogenesis is regulated by the interaction between receptor activator of NFκB ligand (RANKL) and its receptor RANK. RANK is expressed on bone marrow-derived osteoclast progenitors, and its activation upon binding of RANKL is required for differentiation of these progenitors into osteoclasts [10,11]. Osteoprotegerin (OPG), a soluble decoy receptor for RANKL inhibits osteoclastogenesis by interfering with RANKL-RANK interactions [12]. Recently, RANKL has been identified as a potential mediator of cancer-induced bone destruction in humans [13].

Our interest in the role of RANKL/RANK/OPG signaling in deregulation of bone remodeling-associated CaP bone metastases originally led us to examine whether CaP cells in the bone environment expressed these factors. We have shown that normal prostate and primary CaP express RANKL and OPG, and the levels of RANKL and OPG are increased in CaP bone metastases *vs. *those in primary tumors and soft-tissue metastases [14]. CaP cell lines also express RANK and RANKL [14-16], and RANKL was reported to be instrumental in induction of osteoclastogenesis by CaP cells *in vitro *[16]. Soluble RANKL released from CaP cells by MMP-7 was shown to play a role in establishment of CaP bone metastases and osteolysis associated with CaP bone lesions [17]. These data constitute additional evidence of a role for RANK/RANKL signaling in CaP bone metastases.

Results of preclinical studies have shown that inhibition of RANK/RANKL signaling decreases tumor growth and/or establishment and prevented osteolysis in CaP [16,18-22] and other tumor types [23-25] in the bone environment. While these data are promising with regard to treatment of advanced CaP, acquiring additional understanding of the mechanisms of induction of osteoclastogenesis associated with prostate tumors in the bone is a critical step to enable further advances in this area. Cancer cells may cause osteolysis directly by expression of RANKL or indirectly by inducing host production of RANKL. The relative contribution of tumor-versus host-derived RANKL has been difficult to establish, even with human xenograft models, because murine and human RANKL both cause osteolysis in mice. Furthermore, the RANKL inhibitors used to date (OPG and RANK-Fc) [16,18,20,22] or soluble RANK [19] inhibit both human and murine RANKL.

The purpose of this study was to determine whether RANKL expressed by C4-2 CaP cells is directly involved in stimulation of osteoclastogenesis associated with the growth of these cells in the bone environment. We have used a model of experimental CaP bone metastases consisting of direct injection of human C4-2 cells into mouse tibiae and huRANKL MAb, an anti-human RANKL neutralizing antibody to address this question. The use of huRANKL MAb, has enabled us to separate the effect of bone (murine) and tumor (human) RANKL. Our results indicate that the human RANKL expressed by C4-2 cells exerted little or no effect in this system and that RANKL expressed by the host cells within the bone environment is responsible for the stimulation of osteolysis. This implies that CaP cells express factor/s other than RANKL in this murine bone xenograft model that (a) stimulates host cells to produce RANKL and/or (b) directly stimulates osteoclastogenesis.

## Methods

### Tissue culture

C4-2 prostate cancer cells, a subline of LNCaP cells, were purchased from Urocor, Inc. (Oklahoma City, OK) and maintained under standard tissue culture conditions in RPMI 1640 (Invitrogen Corp. Carlsbad, CA) supplemented with 10% fetal bovine serum (FBS) (Atlanta Biologicals, Atlanta, GA).

### Effects of OPG-Fc and huRANKL MAb on huRANKL-induced Ca^2+ ^release in mice

All animal procedures were performed in compliance with the University of Washington and Amgen Institutional Animal Care and Use Committees and NIH guidelines. Initially to characterize the specificity of the huRANKL MAb to bind to human RANKL young male BDF1 mice (4–5 weeks old, n = 5 per group) were injected subcutaneously with 1, 3, or 10 mg/kg huRANKL MAb (AMG 161, Amgen, Inc., Thousand Oaks, CA), 3 mg/kg OPG-Fc (Amgen, Inc.), or PBS. Animals were then challenged twice daily (by subcutaneous injection morning and evening) with either human RANKL (0.5 mg/kg, Amgen, Inc.) or PBS for 6 days. Blood was collected 3 hours after each morning challenge. Blood Ca^2+ ^was measured using a Ca^2+^/pH analyzer (Model 634, Chiron Diagnostics, Halstead, UK).

### Effects of OPG-Fc and huRANKL MAb on systemic TRACP-5b levels in mice

12-week-old female BALB/c mice (n = 5 per group) were injected with a single subcutaneous injection of PBS, OPG-Fc (10 mg/kg), or huRANKL MAb (10 mg/kg). TRACP-5b activity in serum samples collected one hour before (baseline) and 72 h after the treatment was measured using the mouse TRAP™ Assay (SBA Sciences, Turku, Finland).

### Effect of huRANKL MAb on experimental CaP bone metastases

Once the neutralizing activity and specificity of the huRANKL MAb *in vivo *were determined, we set up to investigate the effects of inhibition of human RANKL on osteolysis associated with C4-2 growth in the murine bone. These experiments require use of SCID male mice. We are unaware of any data indicating that bone remodeling would be different between wild-type and SCID mice. Four-to six-week-old male SCID mice were injected with approximately 1 × 10^5 ^C4-2 cells in 10 μL into the tibiae as described previously [26,27]. Three groups of 10 animals each were used: 1) a control group, subcutaneous injection of PBS twice a week; 2) a prevention group, subcutaneous injection of huRANKL MAb (5 mg/kg) twice a week, starting at time of tumor-cell injection; and 3) a treatment group, subcutaneous injection of PBS twice a week until week 3, and then injections of huRANKL MAb (5 mg/kg) twice a week until sacrifice. Blood samples were drawn from animals weekly for determination of serum prostate specific antigen (PSA) levels starting at week 3 after tumor-cell injection, to monitor tumor growth (IMx Total PSA assay, Abbott Laboratories, Abbott Park, IL). All animals were sacrificed 8 weeks after tumor-cell injection. Prior to sacrifice, all mice were radiographed with a Model MX-20 Laboratory radiography System (Faxitron X-Ray Corp., Wheeling, IL). Bone mineral density (BMD) was measured using a PIXImus Lunar densitometer (GE Healthcare, Waukesha, WI). Tibiae with tumors from 5 animals per group were demineralized and embedded in paraffin. The remaining 5 tibiae with tumors from each group were embedded in methacrylate [26,28] for bone histomorphometrical (BHM) analysis. At sacrifice, serum was collected for determination of mouse TRACP-5b activity, using the Mouse TRAP™ Assay, and serum Ca^2+ ^levels, using a PHM 240 pH/ion meter with an ISE25Ca Ca^2+ ^electrode (Radiometer analytical, Lyon, France).

### Bone histomorphometry

Six-micrometer longitudinal sections of undecalcified tibiae embedded in methacrylate and stained with Goldner's stain were used. BHM analysis was performed in the middle of the tibia, in the area 0.525–1.225 mm below the growth plate (n = 5 per group, Skeletech, Inc., Bothell, WA). The percentage of bone volume in tissue volume (%BV/TV), tumor volume in tissue volume (%TuV/TV), trabecular thickness in μm (Tb.Th.), trabecular number per mm (Tb.N.), trabecular separation in μm (Tb.Sp.), the ratio of osteoblast perimeter to bone perimeter as a ratio (Ob.Pm./B.Pm.), and the ratio of osteoclast number to bone surface (N.Oc./BS) were determined. The %BV/TV and TuV/TV was also measured in the whole longitudinal section.

### Immunohistochemistry

Antigen retrieval was performed on 5 μm paraffin sections of tumored tibiae in 10 mM citrate buffer (pH 6) for 20 min at 120°C in an autoclave. The slides were then incubated with 3% H_2_O_2 _for 10 min to block endogenous peroxidase activity, with avidin/biotin blocking solution (Vector Laboratories Inc. Burlingame, CA) for 30 min, and finally with a 5% chicken/goat/horse serum solution for 1 h at room temperature. The tissue was stained with a rabbit polyclonal anti-human RANKL antibody (AB1862, 1/1000 dilution, Chemicon International, Temecula, CA) for 1 h at room temperature. Control slides were incubated with rabbit IgG under the same conditions as the primary antibody. All slides were then incubated with a biotinylated goat anti-rabbit secondary antibody (1:150, Vector Laboratories Inc.) for 30 min at room temperature, and immunoreactivity was detected using the Vectastain ABC kit (Vector Laboratories Inc.) and stable DAB (Invitrogen Corp.).

### Data analyses

Statistical analyses of the results were performed using Prism software (Prism Graphpad, San Diego, CA). Significance of differences was evaluated using paired and unpaired Student's t tests as appropriate, with *p *values ≤ 0.05 indicating statistical significance.

## Results

### Determination of the specificity of huRANKL MAb

#### Effects of huRANKL MAb on human RANKL-induced Ca^2+ ^release in mice

**Figure 1:**
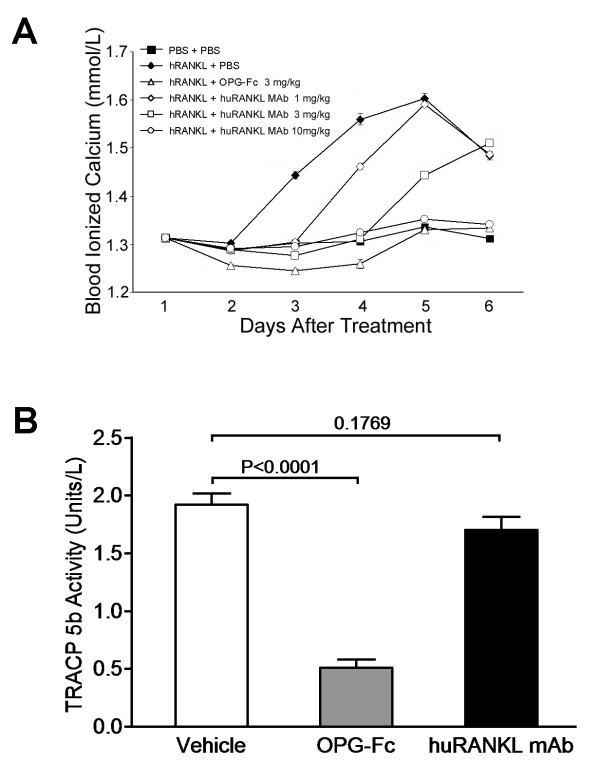
**A**: Measurement of serum calcium levels in OPG-Fc- and huRANKL MAb-treated mice challenged with human RANKL. Young male BDF1 mice (4–5 weeks old) were challenged twice daily (by SC injection, morning and evening) with either PBS (closed squares) or with human RANKL (0.5 mg/kg, closed diamonds) for 6 days. Immediately prior to the first human RANKL challenge, mice were given a single SC injection of either PBS or OPG-Fc (3 mg/kg; open triangle), or huRANKL MAb (1, 3, or 10 mg/kg). Results are expressed as mean ± SE. **B**: Systemic levels of TRACP-5b in sera of mice challenged with OPG-Fc and huRANKL MAb. 12-week-old female BALB/c mice (n = 5 per group) were given a single subcutaneous injection of PBS, (Vehicle), OPG-Fc (10 mg/kg), or huRANKL MAb (10 mg/kg). TRACP-5b concentration was detected in sera one hour before (baseline) and 72 h after treatment (Mouse TRAP Assay, SBA Sciences). TRACP-5b concentration was significantly different between Vehicle and OPG-Fc treated animals (*p *< 0.0001). TRACP-5b concentration was not significantly different between Vehicle and huRANKL MAb treated animals (*p *= 0.1769). Results are expressed as mean ± SE.

In our first experiments, we used normal non-tumor-bearing wild-type mice to establish that the anti-huRANKL MAb effectively inhibits human RANKL-induced increases in serum ionized Ca^2+ ^levels *in vivo*, without showing evidence for the inhibition of murine RANKL. Administration of human RANKL to mice increased serum Ca^2+ ^levels. huRANKL MAb inhibited these effects in a concentration-dependent manner. Administration of high doses of huRANKL MAb (10 mg/kg) completely inhibited Ca^2+ ^release stimulated by human RANKL, returning Ca^2+ ^levels to control levels. OPG-Fc used as a positive control for inhibition of RANKL-induced osteolysis also inhibited Ca^2+ ^release, reducing Ca^2+ ^levels beyond control levels (Figure 1A).

#### Effects of OPG-Fc and huRANKL MAb on systemic TRACP-5b activity in mice

The administration of OPG-Fc to mice significantly decreased systemic TRACP-5b activity in serum of treated mice (*p *< 0.0001). However, huRANKL MAb treatment was unable to inhibit TRACP-5b activity in the serum of mice (*p *= 0.1769) (Figure 1B). After obtaining data that huRANKL MAb inhibits human RANKL, but not mouse RANKL activity *in vivo*, we set out to examine the effects of huRANKL MAb on osteolysis associated with the growth of C4-2 in the bone environment.

### Effects of huRANKL MAb on experimental CaP bone metastases

**Figure 2:**
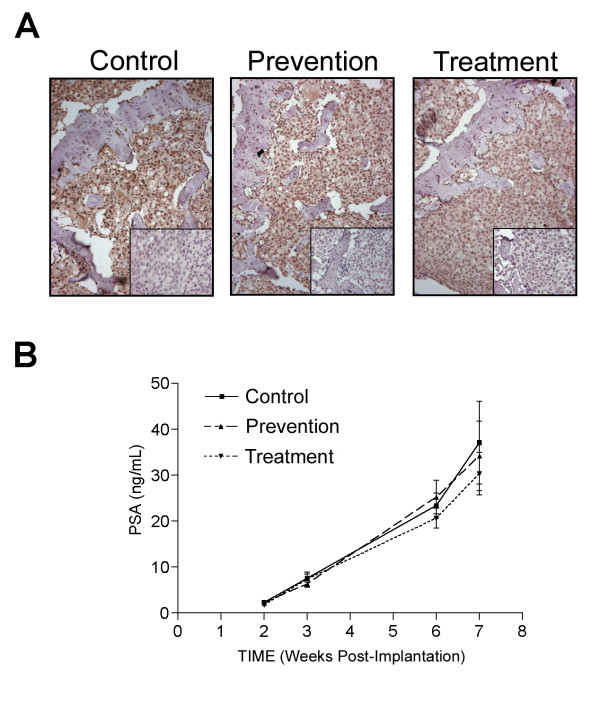
**A**: Immunohistochemical analysis of RANKL in C4-2 tumored tibiae of control, prevention, and treatment groups. Paraffin-embedded C4-2 tumored tibiae of the SCID mice were sectioned and stained for RANKL (negative control staining is shown in inset box). The tumor cells stained positively for RANKL. **B**: Blood was collected to determine serum PSA levels by IMx Total PSA assay. Serum PSA levels were not significantly different between the C4-2 control with PBS injections (control), C4-2 with a concomitant subcutaneous injection of huRANKL MAb (5 mg/kg once a week) at implantation (prevention group), or C4-2 with a subcutaneous injection of huRANKL MAb (5 mg/kg biweekly, starting 3 weeks after implantation of C4-2 cells) (treatment group). Results are presented as mean ± SE.

Once we had established the anti-human RANKL specificity of the huRANKL MAb, we used the C4-2 tibial injection model of experimental bone metastases in SCID mice in combination with the huRANKL MAb to test our hypothesis. All animals had tumor cells growing in the tibiae, as demonstrated by serum PSA levels that were detectable at week 2 after tumor-cell injection and increased thereafter. Expression of RANKL in C4-2 tumors in tibiae was confirmed by immunohistochemistry (Figure 2A). Administration of huRANKL MAb did not significantly alter establishment or growth of tumors in the bone as represented by serum levels of PSA (Figure 2B) and the final tumor volume (Table 1).

**Table 1:** Effect of huRANKL treatment of C4-2 cells on bone morphometry

	BV/TV (%)	TuV/TV (%)	Tb.Th. (μm)	Tb.N. (mm^-1^)	Tb.Sp. (μm)	Ob.Pm./B.Pm. (%)	N.Oc./BS (#/mm)
Control	5.88 ± 0.99	80.48 ± 2.49	31.58 ± 4.83	2.03 ± 0.42	597.24 ± 215.70	5.23 ± 1.03	4.06 ± 0.37
Prevention	6.65 ± 2.31	78.69 ± 2.06	34.27 ± 2.05	1.84 ± 0.50	690.77 ± 188.80	9.51 ± 1.76^a^	4.37 ± 0.71
Treatment	6.75 ± 1.72	77.85 ± 2.99	36.00 ± 4.80	1.79 ± 0.37	633.46 ± 193.00	8.35 ± 2.66	6.32 ± 0.50^b^

Bone histomorphometric analysis was performed in the area adjacent to the growth plate (0.525–1.225 mm below the growth plate: site of injection, n = 4 in control and treatment animals; n = 5 in prevention animals. Two animals were removed from the analysis due to sectioning difficulties. Results are expressed as mean ± SE. Statistical significance was evaluated using student's paired *t *test. ^a ^Control *vs. *prevention (*p *< 0.05). ^b ^Control *vs. *treatment (*p *< 0.05). Abbreviations: %BV/TV, percentage bone volume in tissue volume; %TuV/TV, tumor volume in tissue volume as a percentage; Tb.Th., trabecular thickness in μm; Tb.N., trabecular number per mm; Tb.Sp., trabecular seperation in μm; Ob.Pm./B.Pm., osteoblast perimeter to bone perimeter; N.Oc./BS, ratio of osteoclast number to bone surface.

**Figure 3:**
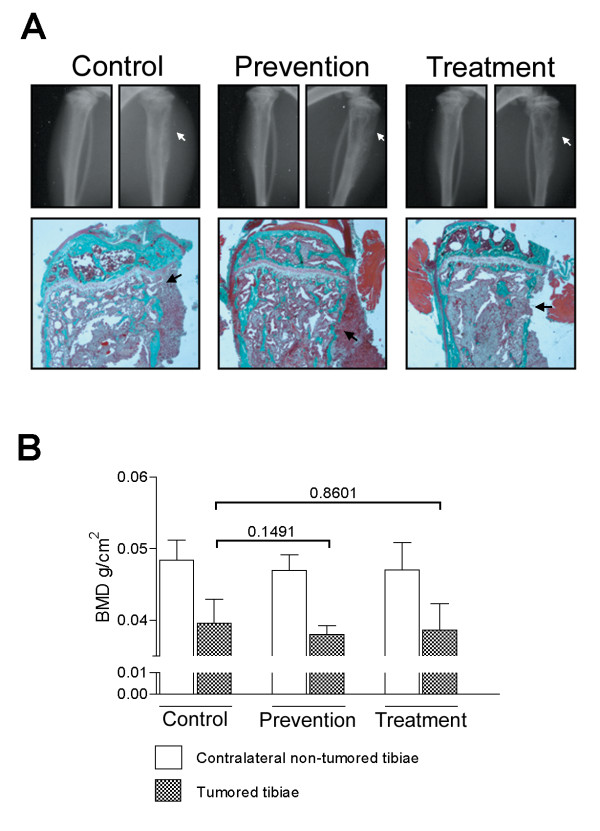
**A**: Characteristics of C4-2 tumor-bearing tibiae. **Upper panel**: radiographs of the right and left tibiae of representative animals from control, prevention, and treatment groups. Arrows indicate areas of the eroded cortical shaft in tumored tibiae of C4-2-treated animals. **Middle panel**: mineralized tibiae were harvested 8 weeks after tumor injection and embedded in methacrylate and 6 μm sections were stained with Goldner's stain. In tumor-bearing animals the tumor replaced the marrow, filling the cavity and degrading the trabecular bone. Arrows indicate areas of the eroded cortical shaft where the tumor has started to extravasate. **B**: Bone mineral density (BMD) was measured 8 weeks after intra-tibial C4-2 injection in SCID mice. There was no significant difference in BMD between control and prevention (*p *= 0.1491) or control and treatment (*p *= 0.8601) animals. Results are presented as mean ± SE.

Radiography of the tibiae was used to assess the impact of the C4-2 tumors on the integrity of the bone (Figure 3A). In the site of injection adjacent to the growth plate, the cortical shaft was eroded and C4-2 tumor cells had replaced the marrow and both the trabecular bone and cortical shaft had been degraded in all groups. In some cases tumor was present outside the cortical shaft (Figure 3A); no significant differences in bone destruction were observed between animals from either groups (Table 1). The destruction of bone associated with the growth of C4-2 cells in the tibiae was demonstrated by decreases in BMD, similarly to our previous data [18,27]; however, administration of huRANKL MAb did not have any significant effects on BMD of tumored or contralateral tibiae (Figure 3B).

Histomorphometrical analyses showed that huRANKL MAb did not alter bone volume (control *vs. *prevention: *p *= 0.5146; control *vs. *treatment: *p *= 0.6467) or tumor volume (control *vs. *prevention: *p *= 0.5920; control *vs. *treatment: *p *= 0.5232) both measured in the region next to the growth plate. Other measurements included trabecular thickness (control *vs. *prevention: *p *= 0.6182; control *vs. *treatment: *p *= 0.6140), trabecular number (control *vs. *prevention: *p *= 0.9855; control *vs. *treatment: *p *= 0.7119) or trabecular separation (control *vs. *prevention: *p *= 0.8950; control *vs. *treatment: *p *= 0.9188). A significant increase in the ratio of osteoblast perimeter to bone perimeter between control *vs. *prevention (*p *= 0.0227) and osteoclast number to bone surface between control *vs*. treatment (*p *= 0.0238) was detected (Table 1). Since the differences in the ratio of osteoclast number to bone surface were detected only between the control and treatment groups, but not control *vs *prevention groups, we speculate that these differences might be associated with the anarchic nature of the bone response to the C4-2 cells and may not be biologically relevant.

**Figure 4:**
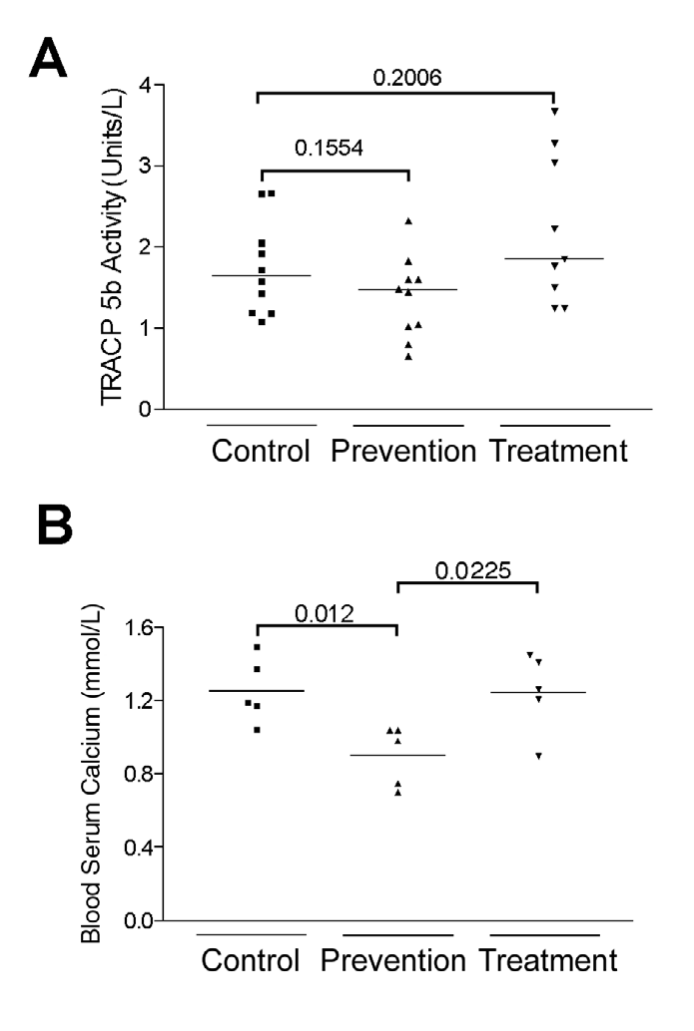
**A**: The tartrate-resistant acid phosphatase assay was performed according to the manufacturer's instructions on mouse serum samples from control, prevention, and treatment groups, using the Mouse TRAP™ Assay measuring TRACP-5b activity (Immunodiagnostic systems Inc.) (n = 10). There were no significant differences associated with huRANKL MAb administration. **B**: Serum calcium levels were measured using a calcium ion electrode 8 weeks after intra-tibial C4-2 injection in the control, prevention, and treatment groups. Serum calcium levels were significantly different between control and prevention animals (*p *= 0.012). Serum calcium levels were not significantly different between control and treatment animals (*p *= 0.9631).

To further evaluate the effects of huRANKL MAb on C4-2-induced osteolysis we measured levels of mouse serum TRACP-5b enzymatic activity. No significant differences in TRACP-5b activity were detected between the groups (Figure 4A). In contrast there were significant differences in serum Ca^2+ ^concentrations between control and prevention (*p *= 0.012), and prevention and treatment (*p *= 0.0225), while no differences were detected between control and treatment groups (Figure 4B).

## Discussion

Even in patients with osteoblastic bone metastases, an osteolytic reaction is also frequently present; however the mechanisms of stimulation of this osteolytic reaction have not been fully elucidated. It has been reported by us and others that CaP cells express RANKL mRNA and protein [14-16,29-33]. Moreover, we have shown that CaP cells in the bone environment express higher levels of RANKL than in primary tumors or soft-tissue metastases [14], and Zhang et al. demonstrated activation of transcription of RANKL in C4-2B cells in the tibiae of mice [34]. The critical role of RANKL/RANK signaling in CaP associated osteolysis has been demonstrated by studies showing that the inhibition of RANKL/RANK signaling decreases tumor associated osteolysis which subsequently results in decreased CaP growth in the bone environment [16,18,20,35]. We have shown previously that administration of OPG or over-expression of OPG inhibits the osteolytic response of the bone to C4-2 cells *in vivo*, clearly demonstrating a critical role for RANKL in this process [18,21]. Similar results were reported in breast cancer and myeloma bone metastases [23,36-38]. In our present study we set out to investigate whether RANKL expressed by human CaP cells in the murine bone environment is involved in direct stimulation of osteoclastogenesis and osteoclast activity or whether the increases in osteolysis are associated with the ability of the tumor cells to elaborate factors that induce host production of RANKL.

In patients RANKL is expressed by both osteoblasts and CaP cells, and it is impossible at present to distinguish the relative contributions of each cell type to the osteolytic component of bone metastases. In the C4-2 tibial injection model of experimental bone metastases, the tumor cells express human RANKL, while the osteoblasts express murine RANKL. The use of a xenograft model and a species-specific RANKL inhibitor provides an elegant model to discern the relative contributions of RANKL derived from tumor cells versus other cell types in the bone microenvironment. This is not possible when using molecules such as OPG-Fc, or soluble RANK-Fc, which each inhibit the action of both mouse and human RANKL. The use of a neutralizing muRANKL MAb might be an alterative way to test this hypothesis; however, we are not aware of an antibody that would specifically recognize murine RANKL and possess neutralizing capabilities.

In our experimental model of CaP growth in the bone, administration of the anti-huRANKL MAb did not significantly inhibit osteolysis associated with the growth of C4-2 cells in the bone, suggesting that the RANKL expressed by these tumor cells is not the driving force for tumor-associated osteolysis in this model. We hypothesize that the interactions between tumor cells and cells within the bone results in increased expression of RANKL on osteoblasts. In support of our hypothesis it has also been reported that DU 145 cells produce soluble factor(s), which increased local RANKL expression and activated both osteoclasts/osteoclast precursors [39]. Other groups have also determined that osteoblasts can upregulate osteoclastogenesis associated factors in response to breast and prostate cancer cells *in vitro *[40,41]. Furthermore, breast cancer cells altered the phenotype of osteoblast cells, causing increased expression of various osteoclast stimulating factors [42]. Potential candidate factors are IL-11 and osteopontin, which were shown to stimulate recruitment and activation of osteoclasts in breast cancer osteolytic metastases, and IL-7 that can stimulate spontaneous osteoclastogenesis in bone metastases [43,44]. Both of these proteins are also secreted by CaP cells [45,46]. IL-11 is an interesting candidate gene, as IL-11 has been shown to increase RANKL expression in bone, and OPG fully prevented IL-11 induced bone resorption [47].

Another anti-human RANKL neutralizing antibody, AMG162 (denosumab, Amgen, Inc.), is a potent anti-resorptive agent that is currently under clinical evaluation as a treatment for cancer-induced bone loss and other bone-loss disorders [48-50]. In patients, the tumor cells and osteoblasts express human RANKL and therefore denosumab will inhibit the actions of RANKL expressed by both cell types. In addition, the use of denosumab has potential advantages as a therapeutic agent over other RANKL inhibitors, such as OPG, because denosumab does not bind to TRAIL and it also has a longer half-life *in vivo *compared to OPG [51-53].

We detected significant decreases in serum levels of Ca^2+ ^in the tumored animals associated with long-term administration of the anti-huRANKL MAb. This effect has not been observed in previous studies (our unpublished data). Since no other alteration consistent with effects on osteoclast number or activity was observed, we hypothesize that long-term administration of the anti-huRANKL MAb might exhibit weak effects on osteoclastogenesis caused by mouse RANKL or that administration of huRANKL MAb inhibits human RANKL/RANK signaling in the CaP cells, altering expression of other factors secreted by these cells that are involved in decreasing serum Ca^2+ ^levels.

As discussed above, we observed no increases in serum Ca^2+ ^levels in animals treated with the anti-huRANKL MAb compared to untreated tumored animals. Nor did we observe increases in TRACP 5b activity after anti-huRANKL MAb administration. As the bone destruction and osteoclastic activity was localized to the right tibia and not systemic, our assays may not have been able to detect minor changes in TRACP 5b activity or serum Ca^2+ ^levels. These assays do suggest, however, that the anti-huRANKL antibody did not have a systemic effect on murine RANKL as osteoclast activity and serum Ca^2+ ^levels did not alter between tumored animals and animals treated with anti-huRANKL MAb. This is a further indication that the antibody anti-huRANKL MAb does not interact with murine RANKL.

We are cognizant that these studies have certain limitations and are subject to alternative interpretations. For example, it could be argued that the dose of the anti-huRANKL MAb (5 mg/kg) resulted in insufficient levels of the MAb in the tumor-bone microenvironment to inhibit huRANKL-mediated osteolysis. However, we do not believe that low levels of the huRANKL MAb are responsible for absence of an effect on osteoclast numbers, since a lower dose of huRANKL MAb (3 mg/kg) inhibited the action of high-dose human RANKL (0.5 mg/kg) in the circulation for four days. It is also possible that the inhibition kinetics of membrane bound and soluble RANKL are different. Prostate tumor cells were shown to produce membrane-bound as well as soluble RANKL [16], but the proportion of these forms *in vivo *has not been determined.

We attempted, but were unable, to demonstrate immunoreactivity of the huRANKL MAb on the C4-2 cells in the bone microenvironment of the tumor-bearing treated animals (data not shown). Not all antibodies recognize the targeted protein after formalin fixation and embedding in paraffin. Therefore the lack of immunoreactivity does not necessarily mean the absence of the target protein. In this particular case, because we have shown expression of RANKL in C4-2 cells in the tibiae using other antibodies, as well as RANKL expression in cells grown *in vitro*, we have concluded that the lack of immunoreactivity of RANKL under immunohistochemical conditions with the huRANKL MAb is due to its inability to recognize the protein in paraffin-embedded tissues. We believe that our selection of this model for our studies is justified for following reasons: 1) we have shown previously that their growth in the bone results in increases in bone destruction; 2) these cells express RANKL; and 3) RANKL is involved in this process.

In this study, administration of huRANKL MAb did not inhibit growth of C4-2 cells within the bone. Since administration of this antibody did not inhibit osteolysis, our results are consistent with the literature showing that inhibition of RANKL/RANK signaling by OPG does not inhibit the proliferation of tumor cells injected subcutaneously in mice [16]. Together these results support the hypothesis that the inhibition of tumor growth by OPG in the bone environment is due to the indirect effects of OPG, through the inhibition of osteolysis and the direct inhibition of RANK/RANKL signaling in tumor cells. However, RANKL signaling has been shown to be important for metastatic spread of tumor cells [54]. These effects were not amenable to study by the methods reported herein, since we injected tumor cells directly into the bone and therefore could not investigate release and trafficking of tumor cells or seeding of tumor cells at secondary sites.

## Conclusion

In conclusion, while RANKL is expressed by CaP cells, and the anti-huRANKL MAb can effectively block activity of human RANKL, our data suggest that tumor-derived RANKL may not be a central player in promoting osteolysis in this model. Our current model supports the notion that C4-2 cells express a factor/s other than RANKL that are ultimately responsible for recruitment and activation of osteoclasts. Based on the ability of OPG to prevent osteolysis and reduce CaP tumor burden in bone, we hypothesize that CaP-derived factor(s) induce the production of RANKL within bone, which then culminates in local osteolysis and the stimulation of tumor growth. Our results warrant further studies to identify factors upstream of RANKL that may be involved in stimulating the osteolytic component observed in this model.

## Competing interests

PJK is a regular full time employee of Amgen Inc., with ownership of stock and stock options. All other author(s) declare that they have no competing interests.

## Authors' contributions

CM performed experiments, data analysis and drafted the manuscript. PJK performed experiments designed the study and modified the manuscript. LGB performed experiments and analysis of the data. RLV was involved in design of animal studies and discussion of the results. EC was involved in the design of the animal studies, discussion of the results, and modified the manuscript.

## Pre-publication history

The pre-publication history for this paper can be accessed here:


